# The conceptual understanding of pediatric palliative care: a Swiss healthcare perspective

**DOI:** 10.1186/s12904-019-0438-1

**Published:** 2019-07-11

**Authors:** Eva De Clercq, Michael Rost, Milenko Rakic, Marc Ansari, Pierluigi Brazzola, Tenzin Wangmo, Bernice S. Elger

**Affiliations:** 1 Institute for Biomedical Ethics, University of Basel, Bernoullistrasse 28, 4056 Basel, Basel, Switzerland; 2 Division of General Pediatrics Pediatric Oncohematology Unit, Hopitaux Universitaires de Geneve Hopital des enfants, Rue Willy Donzé 6, 1205 Geneva, Switzerland; 3 Ospedale Regionale di Bellinzona e Valli, Pediatria Bellinzona, Via Ospedale 12, 6500 Bellinzona, Switzerland

**Keywords:** Oncology, Conceptual barriers, Attitudes, Stigma, Death

## Abstract

**Background:**

Health care providers’ perception of pediatric palliative care might negatively influence timely implementation. The aim of the study was to examine understanding of and attitudes towards pediatric palliative care from the perspective of health care providers working in pediatric oncology in Switzerland to promote the timely implementation of pediatric palliative care.

**Methods:**

Five mixed focus groups were conducted with 29 health care providers (oncologists, nurses, psychologists, and social workers) at five Swiss pediatric oncology group centers. The focus group interviews were analyzed using thematic coding.

**Results:**

Most participants associated pediatric palliative care with non-curative treatment. They regularly reported difficulties in addressing palliative care services to families due to the strong stigma surrounding this term. They also thought that the notion of palliative care is very much linked to a policy context, and difficult to reconcile with children’s everyday life. To overcome these obstacles many participants used synonyms such as comfort or supportive care. A few providers insisted on the need of using palliative care and reported the importance of positive “word of mouth”.

**Conclusions:**

The use of synonyms might be a pragmatic approach to overcome initial barriers to the implementation of palliative care in pediatrics. However, this tactic might ultimately prove to be ineffective as these terms might acquire the same negative connotations as palliative care. Positive word-of-mouth by satisfied families and healthcare providers might be a more sustainable way to advocate for pediatric palliative care than replacing it with a euphemistic term.

## Background

Since its introduction in 1975 the term palliative care (PC) has been subject to fluctuations in meaning [1–5]. For almost two decades the term has been used interchangeably with hospice, end-of-life or terminal care [3]. In 1990 the World Health Organization (WHO) shifted away from the “end-of-life” mindset by stating that palliative care is applicable earlier in the course of illness, in conjunction with anticancer treatment. This definition was further amended in 2002 when PC was uncoupled from prognosis and the target population was broadened to include patients facing a life-threatening condition [2, 5].

The current gold standard approach in PC, as defined by the World Health Organization (WHO), consists of the *concurrent* administration of curative treatment and PC with attention to patients’ physical, psychological, social, and spiritual needs. The definition provided by the WHO [6] is said to be a philosophical, rather than a dictionary definition [7] as it does not just report what “palliative” literally means (“the alleviation of suffering”), but invokes certain values (e.g. patient-centredness, holism and multidisciplinarity) that are aimed to guide action and improve practice [1, 8]. The principal aim of PC is to maintain the quality and the meaningfulness of life for both patients and their families. Unlike hospice care, PC is not limited to terminal care, but is appropriate for patients in all disease stages [6].

Within pediatrics and in pediatric oncology the early implementation of PC has been widely endorsed [9, 10] as it has been associated with improved survival and quality of life [11–17]. According to the “Cancer Pain Re-lief and Palliative Care in Children” document [18], pain management for children should start *at diagnosis* and continue throughout the course of illness alongside curative treatment. Despite these recommendations, patterns of late referral continue to persist in pediatric oncology [16]. As a result, many children – like many adult patients – do not benefit from pediatric palliative care (PPC) or at least not in a timely manner [19]. Clinical reality appears thus to lag behind the paradigm shift made within PPC guidelines [19–21]. This suboptimal integration of PPC leads to a paradox: if the early introduction of PPC is beneficial to the child’s and family’s well-being, then why is there such reluctance to implement this principle in medical practice [22]?

Studies exploring barriers to PC have focused mainly on PC in adult healthcare [23–31]. Given that the group of pediatric patients with PC needs is considerably smaller compared to the adult patient group, fewer studies, with some noteworthy exceptions, have focused on barriers to PC in pediatrics [20, 32–34] and evidence specific for pediatric oncology is rather limited [35]. Of these studies, the majority have used quantitative methods and/or focused on the perspective of a single type of pediatric healthcare provider. In all these studies family and patient reluctance to accept PPC is often cited as an important (perceived) barrier to early integration [13, 32, 36] and related to misconceptions of PPC goals due to its equation with death and dying [37].

Still, a recent survey study on pediatric cancer patients’ and parents’ attitudes toward early integration of PPC in oncology seems to debunk the myth that parents and children are not ready to integrate PPC [13]. Interestingly, the study found that for the majority of children and parents who participated in the study, the term “palliative care” was *unknown*. In the survey, the researchers described the PPC team as “a group of clinicians with expertise in symptom management and a goal of improving quality of life”. These findings suggest that patients’ and parents’ attitudes toward PPC integration is influenced by the way in which PPC is explained [28] and that (in) adequate understanding of PPC by healthcare providers may bias families’ attitudes and decisions towards the integration of PPC.

In Switzerland the knowledge gap regarding palliative care among lay people is still great [38] and awareness of children’s unique PPC needs is scant compared to other countries like the UK [39]. Those who are familiar with the notion of palliative care associate it with a type of care that focuses on quality of life when faced with an incurable illness. A recent retrospective analysis of medical records of deceased pediatric patients has shown that late and non-referrals are still very common in the Swiss oncology setting and that a dualistic model of curative and palliative care continues to prevail [40]. Since health care providers’ perception of PPC might influence that of parents and children, the present study aimed to examine the understanding of and attitudes towards PPC in Switzerland from the perspectives of health care providers working in pediatric oncology centers.

In order to gain a richer, more detailed account of staff members’ attitudes toward PPC and to identify possible socio-cultural factors that influence their perceptions, we conducted focus group discussions with various stakeholders in the field of pediatric oncology: oncologists, nurses, psychologists, and social workers. The study goal is particularly important given that health care providers might *unwittingly* associate PPC with end-of-life care even if they claim to support the early introduction of PPC [32]. Thanks to their interactive nature, focus groups provide access to data that might be less easily obtained through surveys or individual interviews as some thoughts can only be probed within a group context [41]. Furthermore, in order to develop appropriate interventions that improve PPC provision within the field of pediatric oncology, it is important to listen to different care providers, especially in the Swiss context where PPC is usually provided by the primary oncology team rather than by PPC specialists. Since divergence of opinions on PPC is not uncommon among team members and might lead to an inconsistent message about PPC in interaction with families [42], we intentionally used a *mixed* focus group approach. Finally, it is increasingly recognized that qualitative insights play an important role in closing the policy-implementation gap [43–45], this is particularly relevant for the PPC context where (conceptual) implementation barriers continue to persist despite the development of educational programs, PC’s increased focus on quality of *life* (rather than on death) and its overall beneficial outcomes.

## Methods

This study is part of a larger project on end-of-life decision-making in pediatric oncology where (a) surveys were carried out with physicians and parents and b) interviews were conducted with children suffering from childhood cancer, their parents, and physicians; and (c) a retrospective data collection from medical records was performed [46–49]. For this qualitative part of the study, mixed focus groups were conducted with health care providers at five of the nine Swiss pediatric oncology group centers (SPOG): three out of six centers in the German-speaking part of Switzerland, and both of the two centers in the French-speaking part. The remaining four SPOG-centers decided not to participate in the focus groups due to lack of time. Approvals were obtained from the respective cantonal research ethics committees.^1^

For each SPOG a reference person was identified who helped with participant recruitment and focus group scheduling. The reference person informed all members about the overall aim of the focus groups and their confidential nature. Upon consent of the participants, the recruiter then gave the research team a list of team members who had expressed interest to participate. Each of these persons received the participant information sheet from a member of the research team via email, immediately after recruitment and then again (as a reminder) some days before the actual date of the focus group discussion to give participants enough time to read over the participant sheet. The research team sent a request to all participants to find a common time to carry out the focus group discussions. Since initial recruitment happened with the mediation of a reference person it was difficult to establish how many persons refused to participate (mainly because of lack of time).

Focus groups were carried out by the first and second author between August 2016 and June 2017 at the SPOG centers at a time agreed upon by all participants. At the time of the interviews, the first author was a postdoctoral researcher with a background in philosophy and ethics. The second author was a doctoral student with a background in psychology and ethics. Both researchers had experience with conducting interviews. The total number of participants for each discussion varied between 4 and 8 healthcare providers. Oral informed consent was sought from all participants prior to the start of the focus group and registered upon consent. From an ethical point of view, for minimal risk research involving interviews studies with health care professionals whose data (transcripts or questionnaires) are anonymized, oral consent and active participation are ethically considered sufficient and proportionate. Furthermore, in Switzerland interviews with health care professionals (not patients) are outside of the human research act and do not require ethics committee approval. To make sure that our experts were clearly informed, at the beginning of the discussion, the moderator briefly restated the purpose of the overall project, their role in the project and allowed participants to ask questions.

A semi-structured interview guide framed each focus group discussion. The guide was built on the data obtained from a retrospective review of medical records of children who died at the SPOGs between 2008 and 2014 [49] and on the experiences of the research team during prior phases of the overall project. In order to fine-tune the questions the interview guide was evaluated by a collaborator working at a SPOG. Questions included information about (a) the participants’ personal understanding of PPC, (b) the institutional attitude towards PPC, (c) discussions and communication processes with parents and children regarding PPC, (d) perceived obstacles to the implementation of PPC and (e) institutional referral practices. Questions on topics (c) to (e) were discussed with reference to a specific recent case that the team encountered. Most of the data presented in this paper derives from the questions related to topics (a), (b) and (d) as they deal with *conceptual* barriers. The other topics will be analyzed in a future manuscript.

The five focus groups lasted between 90 and 120 min. One team member was the moderator; another team member was the co-moderator who took notes, kept track of time, and helped in asking follow up questions. To facilitate qualitative analysis, all the discussions were tape-recorded and transcribed verbatim in the language of the interview (German or French). Transcripts were returned to participants for revision.

The 5 focus group transcripts were checked for accuracy by three researchers and transferred into the qualitative analysis software MaxQDA (version 12) to support the analysis process [50]. Three authors independently analyzed the transcribed data by reading the interviews several times. After a close line-by-line analysis of the transcripts, provisional categories were identified by each of the three researchers. In a next step – to ensure consistency in the analysis of the data – the three team members discussed their respective categories and reached an agreement about the coding scheme and superordinate themes across the different focus groups were developed. In a final step, the first author reviewed the group level thematic taxonomy through the eyes of each individual participant to see which themes represented and did not represent the individual’s account. For this purpose, everything a single participant said was, first, marked in a specific colour and then re-read and compared to the themes discussed by the group as a whole. In this way, both differences and commonalities among participants were identified together with the overall context that triggered their claims. This step was important given that the focus groups were mixed and we wanted to do justice to the concerns of each individual and provider type. Saturation of data was reached after the 5 focus groups.

## Results

A total of 29 persons (working at 5 different SPOG centers) participated in this study: 14 nurses (among which 2 nurses specialized in the field of PPC), 10 physicians, 4 psycho-oncologists/psychiatrists, and 1 social worker (see Table 1).

**Table 1:** Participants’ characteristics (*N* = 29)

All participants	N (Percentage) / Mean (SD)	
Gender (women)	21 (72.4%)	
Experience in years	14.9 (8.9)	
Professions	Experience in years Means (SD)	Gender in percent (women)
Nurses (*n* = 14)	16.3 (9.8)	92.9%
Physicians (*n* = 10)	16.1 (8.4)	30%
Psycho-oncologists / Psychiatrist (*n* = 4)	7.5 (4.1)	100%
Social worker (*n* = 1)	12	100%

**Fig. 1:**
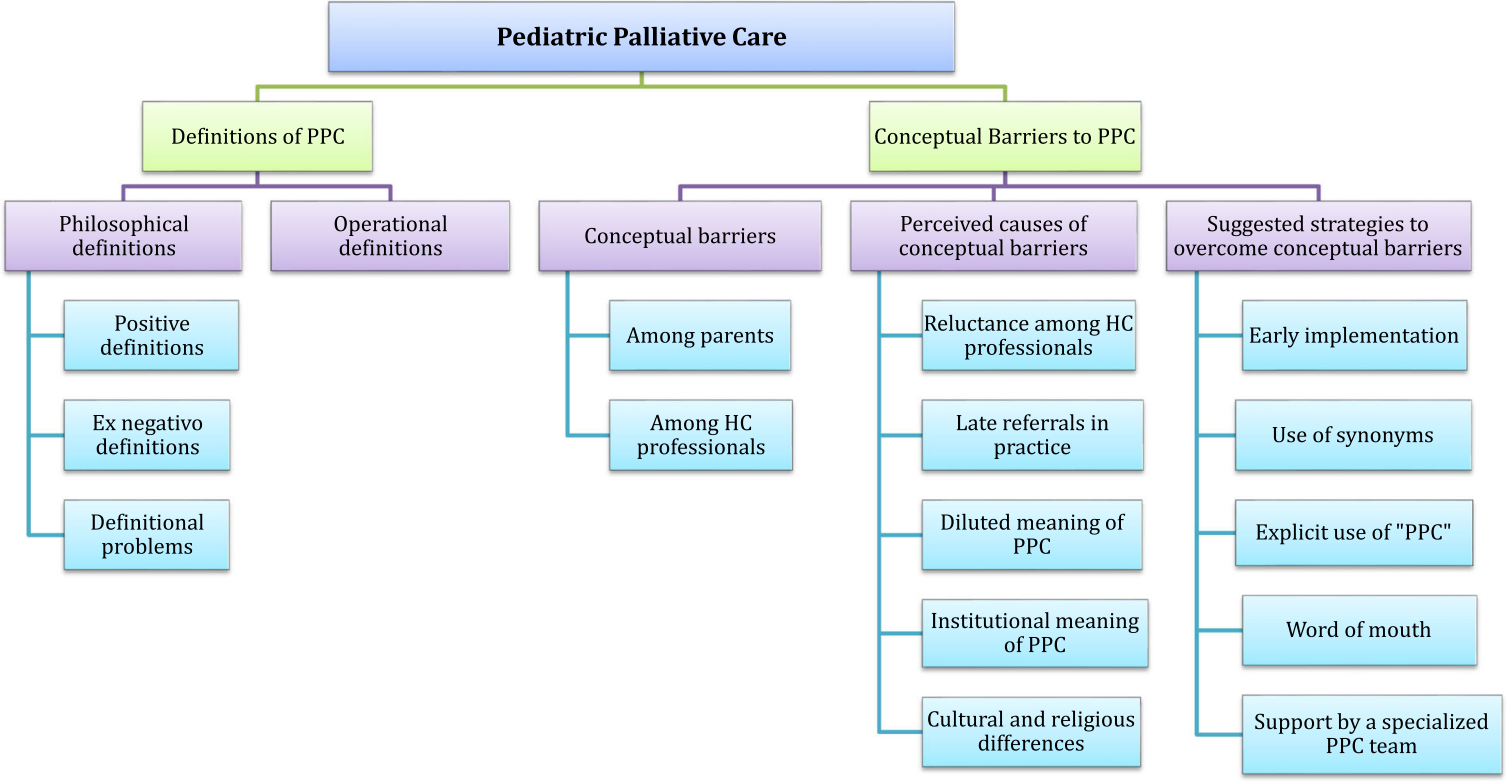
Themes and subthemes

Analysis with regard to the conceptual understanding of PPC by pediatric oncology care providers identified 2 major themes (and several subthemes): (1) Definitions of PPC and (2) Conceptual barriers, their causes & possible solutions. To improve clarity, the themes and their respective subthemes are presented in Fig. 1.

To illustrate the reported results, representative quotes were taken from the various interviews using pseudonyms for the participants as well as the center. All quotes were translated from German and French into English, and translations were checked for accuracy by two authors. Quotes will be presented in tables so that all three perspectives on a particular topic are easier to compare and also to provide an insight into the richness of experiences and opinions gathered.

### Definitions of PPC

#### Philosophical definitions of PPC

There was overall consensus on the common principles and values of PPC. In fact, most participants listed features that are essential to the gold standard of PPC: a family centred and active approach to care, offered by a multidisciplinary team and incorporating physical, psychological, social and spiritual needs to ensure the best possible quality of life throughout the illness trajectory (see Table 2.1, section a).

**Table 2:** Definitions of PPC

1. Philosophical definitions	
a) Positive definitions	i.) PPC is gold standard (WHO) For me palliative care means comfort care, holistic care, it means taking care of the child and his/her family in a physical and psychological sense; total care of the entire family. (*Nurse, speaker 2, center 1*) It really means «not to abandon» … to provide palliative care is something very active, to stand by all the life projects until the very last second. (*Nurse, speaker 1 with PPC specialization, center 2*) The most important thing is really the quality of life and (...) to create a network for families, for the whole family (...) important is also to collaborate and to have a multidisciplinary team. (*Nurse, speaker 3, center 3*) For me it is important that the persons who cared for the patient before do not just disappear and leave the patient alone (…) but that the contact remains until the end and even beyond the end, ideally. (*Physician, speaker 1, center 5*)
b) Ex-negativo definitions	i.) PPC is not end-of-life care To me it is quite clear that palliative care is something for the child (…) within a comfort approach and with the aim of increasing the child’s quality of life. But it does not necessarily imply the imminent end-of-life. (*Nurse, speaker 4, center 2*) End-of-life care starts normally during the last 4 weeks, but PPC starts of course much earlier. (*Physician, speaker 1, center 4*) ii) PPC is not curative treatment [PPC] is the total care of a pediatric patient from the moment one knows that the oncological disease is incurable. (*Physician, speaker 5, center 1*) In some other areas of PC it is different – in the case of a chronic condition the child cannot be cured anyway. In oncology, however, we always start with a curative treatment, but then there are situations (…) in which we say: we cannot cure the child (…) and then there is a switch to palliative therapy, which can take a long time, but then we know that the child will die either of the illness or the treatment. (*Physician, speaker 1, center 3*) The shift from curative to palliative, I think that in Switzerland that is very different from the UK where palliative care teams are integrated from the time of diagnosis (…) until there is still hope for a cure, that they will find a treatment ... Here [in Switzerland] they will not consider them [patients] palliative. (*Nurse, speaker 2 with PPC specialization, center 2*). iii.) PPC is not an abrupt shift It’s true that from a medical point of view there is never an abrupt shift from a curative to a palliative approach, there is always a transition phase (…) often the family and the patient, but maybe physicians too need such a transition time before coming to terms with the fact that the child will not heal. It’s not something on/off. (*Physician, speaker 5, center 1*) The decision-making regarding the shift from curative to palliative treatment, is a process (…) in which one goes back and forth, in which one is not fully certain … it is not a fixed point in time. (*Psycho-oncologist, speaker 5, center 4*)
c) Definitional problems	i.) Disagreement on the meaning of PPC I do not agree. For me curative and palliative are not in opposition, or consecutive, they are often rather concurrent, especially in the case of our patient group [pediatric oncology] where once the one aspect then the other one can be more important. (*Physician, speaker 3, center 4*) ii.) Acknowledgement of various definitions I believe that you can understand many different things under palliative care and that is precisely the problem. (*Physician, speaker 2, center 4*)
2. Operational definitions of PPC	
	i.) Uncertain timing due to definition The shift from curative to palliative is not that clear. From when can we say that a person is in palliative care? In practice this passage is not so clear to me. On the other hand what palliative care stands for I think is very clear here [to professionals in the hospital] (*Social assistant, speaker 2, center 2*) ii.) team discussions due to unclear definition I still find it really difficult to say that now a patient is palliative or not. (…) I really notice that nurses and doctors often have a different view on the whole. Sometimes we do not immediately find a consensus. Maybe the concepts are not clear (…) I remember the last big roundtable we had over a difficult case in order to all have the same understanding and to bring in ideas in order to know: “what next”? (*Nurse, speaker 4, center 5*) It [PPC] was introduced quite late in the service (…) in some situations the children felt more and more uncomfortable, they had a poor quality of life and the nurses were the ones who shouted: “Help!” (…) (*Nurse, speaker 2, center 1*). The individuality within the team, the different values, the different attitudes towards life, are often not explicitly made (…) what remains unsaid can create barriers (…) it can lead to fractions or different fronts, without it being made explicit (*Psycho-oncologist, speaker 5, center 4*) It’s [PPC] about human perceptions and everyone has their own perception and suddenly, when we change the therapeutic goal everyone is put into confrontation (*Physician, speaker 5, center 1*) iii) need to overcome conflicts Consensus is absolutely necessary (…) you have to try to find it. Which way do you want to go, how do you want to go there? (…) perhaps you need to get together two or three times. Even if you want to avoid conflicts (…) you have to face them (*Physician, speaker 1, center 5*).

In addition to these definitions that describe what PPC is or should be, many participants also provided “ex-negativo” definitions, that is, they used expressions to describe what PPC is *not* (see Table 2.1, section b). Most providers insisted on the fact that PPC is not the same as end-of-life care or care provided when death is imminent. However, at the same time the majority of the participants insisted on the fact that *within the Swiss oncology* context, it is best to provide PC when there is no response to curative treatment. Among them many believed that understanding palliative care as non-curative care was crucial in order for the team “to know where they stand” and to be able to communicate with the parents in a clear and honest way (see Table 2.1., section b, ii). Despite this shift, various participants insisted that this is not an abrupt one, but a gradual process which allows everyone involved to accept the fact that the child is dying.

There was one participant who explicitly disagreed with the description that PC starts when there is no hope for cure. Within this regard it is important to highlight that participants were aware of the existence of other, often contradictory, definitions of PC (see Table 2.1, section c).

Although most participants agreed that the provision of PPC is closely connected to the absence of cure, this rather broad understanding of PPC raised important challenges when implemented in practice.

#### Operational definitions and operationalization of PPC

Many participants acknowledged that clear guidance was missing on an operational level (see Table 2.2) and that this led to divergence of opinions among team members, especially with regard to the question of *when* to introduce palliative care. A physician reported (speaker 5, center 1): «There is always, or very very often, a moment when –I do not know how many times I have nurses heard saying: “but why do they [physicians] continue?”». Nurses recounted that they sometimes have the feeling that physicians push the boundaries too far and implement PPC too late. A nurse (speaker 4, center 1) reported how upset she was when she forbade a young patient to eat the sausage of which he was so fond, while knowing that he might only live for another 3 months: «We are the ones who enforce the [food] prohibition (…) that’s maybe why we start asking ourselves questions more quickly».

On the other hand, nurses acknowledged that they do not always have the same medical information as the doctors, and that this can create divergence. They thus noted that it is important to sit together so that everybody is on the same page. Some participants expressed the concern that these disagreements might be due to confusion about the meaning of PPC: «Sometimes there is no agreement because the definitions are unclear» (nurse, speaker 4, center 5).

### Conceptual barriers, their causes & possible solutions

#### Conceptual barriers among parents and professionals

Many participants – across the different provider types – were convinced that parents are reluctant to start PPC because they associate it with “death and dying”, “loss of hope”, and “giving up” (see Table 3.1, section a). According to a social assistant (speaker 2, center 2) the stigma surrounding PC is so substantial that «the term “palliative” is the biggest enemy» when addressing families. Some participants acknowledged that they also often “struggle” with the concept and have difficulty accepting the next phase in the child’s illness course (see Table 3.1, section b).

**Table 3:** Perceived conceptual barriers & their causes

1. Perceived conceptual barriers	
a) Among parents	i.) PPC equals death, giving up, loss of hope There are parents where you have the feeling that they do not consent to the palliative care process because they think: «I am giving up on my child». (*Psycho-oncologist, speaker 4, center 3*) For many families “palliative” means “death”, whereas palliative care does not mean that you will die. (*Nurse, speaker 2, center 1*) The difficulty might be to address the issue of death, but it is also like (…) if we abandon all efforts. For parents this is hard, because they perceive it as “we give up” and it does not meet their expectations. (*Psycho-oncologist, speaker 7, center 2*) The last case was a patient who medically speaking was in very bad shape (…) the mother fought with all her strength against the slow change of [treatment] course [from curative to palliative]. She had the impression that we were denying the boy the chance to heal. (*Physician, speaker 7, center 5*)
b) Among HC professionals	i.) Struggle to accept a next phase There is much work to do regarding the wording; many things belong to us, to the caregivers, our difficulty to accept that we pass from a curative to a palliative phase. (*Psycho-oncologist, speaker 7, center 2*) The concept [of PPC] is one thing, it is a bit the rational part, like safeguards and structures it offers support, orientation and security. On the other hand, you have the attitude of the department … how we [team members] *actually* experience palliative care (…), what we live *(Psychologist, speaker 5, center 4)*
2. Perceived causes	
a) Reluctance among professionals	i) HC professionals’ fear of PPC If there is no pain yet or something that leads to palliative care (…) I feel it as an obstacle myself. We are afraid of pronouncing the word and at the same time we do not know how to tell it differently. (*Physicians, speaker 5, center 2*)
b) Late referral practice	i) PPC as the grim reaper We only talk about PPC at the last minute; it’s like we sign the child’s death warrant (…). So maybe if we were introduced before, it [PPC] would not have the same effect on the family (*Nurse with PPC specialization, speaker 1, center 2*) If we take the definition of palliative care, and ask ourselves whether death is probable, unavoidable within “6 months”, so the speak (…) then I think we do not anticipate the situation enough and then all of a sudden it declines very quickly most of the time. *(Nurse, speaker 1, center 1)*
c) Diluted meaning of PPC due to integrative approach of WHO	i) No clear distinction leads to confusion It’s very hard to have a discussion and to state “not everything is lost yet” and at the same time mention “palliative care”. (*Physician, speaker 5, center 2*) ii) Total care is always already provided Holistic care is there already (…) that’s why there is confusion, I think. But ok, that is the WHO definition. Comprehensive care is present throughout the illness course, whether it is in the palliative or curative phase. (*Nurse with PPC specialization, speaker 1, center 2*) The concept [PPC] disturbs me (…) I think we have been providing medical care in a humane way for centuries and I do not think (…) care has to be renamed (…) the patients must receive best supportive care from the beginning until the end of life. (*Physician, speaker 7, center 5*) What would be the added value, which is currently missing, if one would integrate it [PPC] from diagnosis onward? (…) there is already a total care approach (…) I think there is not necessarily an advantage. (*Physician, speaker 3, center 2*)
d) Institutional meaning of PPC	i.) The term PPC is not related to the life-world of children I think the word “palliative” (…) is inadequate in pediatrics because if we place a person of 50 or 60 years old in palliative care, we know that the person will die at an age close to the natural age of death with a palliative care treatment of 10–20 years depending on the type of illness. In the case of a child (…) we will not be able to reach the natural limit. (*Nurse, speaker 4, center 1*) We knew that talking about palliative care might constitute a barrier to certain families, but for the public health sector who finances us, it was a must (…) we needed to call ourselves [the service] “palliative”. (*Nurse with PPC specialization, speaker 1, center 2*) An important topic is the difference between the life-world of adults and the life-world of children (…) when one in Switzerland talks about palliative care or about its political or infrastructural aspects then one has to be aware that the child is not taken into consideration. (*Physician, speaker 2, center 4*)
e) Religious & cultural differences	Something that makes it difficult to address palliative care is that 80% of the immigrant population comes from far away (…) with many different cultural backgrounds (…) What does palliative care mean for a Swiss person, but also in the broader sense in the world. Culturally speaking, what does it [PPC] mean? (*Nurse, speaker 4, center 1*)

#### The perceived causes of conceptual barriers

Some participants acknowledged that their own attitudes towards PPC might negatively influence those of families’ and further compromise timely implementation of PPC (see Table 3.2, section a).

Another important barrier that was cited (mainly by nurses) for the wrongful association between PPC and death was that PPC is still implemented relatively late in the course of the illness, in response to a bad prognosis or when children’s quality of life becomes very poor (see Table 3.2, section b). On the other hand, some participants seemed to be concerned that introducing PPC too early; at the time of diagnosis – as set out by the WHO guidelines – risks diluting the meaning of PPC (see Table 3.2, section c). If PPC is uncoupled from the actual dying process and the non-curative phase, then «we are all in a palliative situation» (physician, speaker 3, center 2) insofar we all will die 1 day and this will cause confusion. Some were concerned that by uncoupling PC from dying, one risks marginalizing an important component of PPC, namely bereavement care. «There is little notion (…) in oncology of bereavement care which is an essential part of palliative care» (Nurse, speaker 2, center 1). Others reported that all-round care is provided from the moment the patient enters the hospital, and wondered what difference introducing PPC earlier would really make (see Table 3.2, section c).

As well as issues related to the timing of PPC, participants also argued that the notion of PPC is very much linked to an institutional, political and professional context, but that this PC philosophy is difficult to reconcile with children’s every day life: «In the child’s personal context what does that mean, palliative care?» (physician, speaker 3, center 2) Various participants associated PC with adult, and in particular elderly, care and emphasized that also on the policy level PC was rarely addressed with regard to children (see Table 3.2, section d).

A final cause has to do with the cultural and religious background of patients and their families. When parents’ background significantly differed from traditional Swiss culture, participants frequently reported difficulties with introducing and providing PPC. In many cases, it was the presence of pain which finally convinced the parents to implement PPC, but this was often at a rather late stage (see Table 3.2, section e).

#### Suggested strategies to overcome the conceptual barriers

The participants provided several strategies to help overcome the conceptual barriers. Some of them suggested introducing PPC earlier so that this type of care would be part of the “landscape” and not be considered to be the “grim reaper” (Nurse, speaker 1 wit PPC specialization, center 2). Timely implementation of PPC was believed to promote a more natural transition and take away the a priori negative connotation of PPC (see Table 4.1, section a).

**Table 4:** Strategies to overcome conceptual barriers

1. Strategies to overcome conceptual barriers	
a) Early implementation of PPC	i) Timely introduction Perhaps the fact of intervening sooner would create less fear. When people hear “palliative care” they immediately think: “Ah that means: death”. (*Nurse, speaker 8, center 2*)
b) The use of synonyms	ii) Euphemisms I like the term “accompagnement” in these situations (…) it means “being with” (…) “palliative” evokes, in my opinion, something a priori negative and I do not feel comfortable using it. (*Nurse, speaker 3, center 1*) For me, more than using this word [PPC], it’s more about contextualizing it together with the parents and the child. I think that the child and the parents have a different view on the situation. We must try to make them understand that knowing that a disease is incurable does not mean stop giving care, but entails a different way of caregiving (…) one should use the word “comfort care”, for me that is more appropriate (*Nurse with PPC specialization, center 2*)
c) Explicit use of the term PPC	i) De-bunk the stigma We need a common language (…) You have to call a cat ″a cat″ (…) among the general public there is maybe a kind of ″mystification″ of palliative care, in the sense of palliative equals death, but it is our task as professionals to finally inform the public on the federal level and the rest. (*Nurse, speaker 1, center 1*) ii) Bring clarification to family & team The concept (…) should bring clarification to the treatment team and the patient. Therefore I actually understand palliative care as non-curative care. That is a crucial point for me, which I then need to discuss with the parents so that we know where we stand. (*Physician, speaker 2, center 4*) We on the nursing level use the word (…). We say: «the doctors have talked with the parents; we are in a palliative care situation now». I think for us this word signifies a change of attitude in the perception of the type of care we will provide. (*Nurse, speaker 4, center 2*)
d) “Word of mouth”	i) Family satisfaction We deprive ourselves a little of the opportunity to give them a chance (…) In the family room I see how much families speak to each other. Every time a family has really benefitted and been satisfied, well, this information goes around. Families speak a lot among them *(Social assistant, speaker 2, center 2)*
e) Support of specialized PPC team	i.) Benefits of specialist PPC team I think we would benefit to have a systematic support [external PPC service] (…) to bring a bit of order in the discussion, in the ideas, in the emotions when preparing to pass from curative to palliative care. (*Nurse, Speaker 3, center 1*) ii.) Drawbacks of specialist PPC team There are different models in Switzerland and in the world, also in pediatrics, on this subject matter, with some hospitals having specialized teams in the background (…) I think that it is not desirable (…) there are not so many cases in oncology (…) it would be an exaggeration to have such a team (…) it would also create certain problems. (*Physician, speaker 1, center 4*)

Several healthcare providers suggested rebranding PPC by using a synonym which is more easily understood and less stigmatized, such as comfort care, best supportive care or “*accompagnement*”^2^ as these words do not directly relate to death and dying. They believed that these more commonly used terms better expressed the caring goal of PPC (see Table 4.1, section b).

A few participants, however, insisted on the importance of using “palliative care” because they feared that the use of a euphemism would not really help overcoming the stigma associated with the word. They further emphasized that it was healthcare providers’ task to promote the values of PPC among policymakers and the general public. The use of the term “palliative care” was also said to be crucial to give a clear indication of the patient’s overall treatment and care goals and thus to have a common language and understanding within the team (see Table 4.1, section c).

A few participants also reported about the importance of “word of mouth”: families who are satisfied about PPC can help overcoming the taboo surrounding this type of care and become PPC advocates (see Table 4.1, section d).

Finally some care providers highlighted the importance of having an external specialized PPC team as this could offer an additional perspective on the situation and provide support to the primary team, who might be too emotionally involved to start PPC in a timely manner. On the other hand, in some centres, concerns were raised about whether different teams could successfully work together (see Table 4.1, section e).

## Discussion

Early PPC is encouraged by various international oncology organizations as it has been associated with improved symptom management and quality of life of both children and their families [21]. Still, to date, many patients who could benefit from PPC services do not receive them, or at least not in a timely manner [51]. Conceptual confusion has been identified as an important barrier to adequate implementation of PPC. Given the influential role of healthcare professionals on families’ decisions, this study offers a unique insight into the personal understanding of and attitudes towards PPC among various types of pediatric oncology providers. There is only a small body of literature on barriers to PC in pediatrics and most of the existing studies have used quantitative research methods. However, in order to better understand the “paradox” in PPC – the persistence of late and non- referrals despite PC’s beneficial impact – qualitative research might be more appropriate as it enables a more complex insight into people’s opinions and motivations.

The study results show that most participants recognized the important value of PPC and had good knowledge of its core principles and objectives. The interesting finding is that although they clearly distinguished PPC from end-of-life or terminal care, many of them – across the various provider types – insisted that, within the pediatric oncology context, PPC is best provided when curative treatment is no longer an option. Thus, although the WHO guidelines embrace an integrative approach from the time of diagnosis, most participants defined the target group for PPC in a much more restrictive sense. However, they also emphasized that the transition from curative to PPC is not abrupt, but is actually a rather gradual process during which both families and healthcare providers slowly but steadily become aware of the need to re-direct the care provision. Understanding PPC as non-curative was considered necessary to generate this change in attitude. Some participants expressed the concern that “mixing” the two care approaches might cause confusion among both family and staff members and thus be counter-productive.

While most participants supported PPC principles, they were worried about the lack of guidance on implementing them within clinical practice. Timely integration of PPC continued to pose a problem on the operational level and led to conflicts among the team members. Due to their daily contact with patients and families at the bedside, participants believed that nurses are more pro-active than physicians in encouraging PPC. Various interviewees recognized their own personal difficulties with the transition of treatment goals and welcomed the intervention of a specialized PPC team to lessen the burden. However, not all participants were in favour of such a specialized PPC team. Some believed that the primary oncology team could offer all the necessary PPC and seemed to be concerned about possible interpersonal conflicts. Research has shown that such conflicts are not unusual as the non-hierarchical structure of PPC tends to challenge the traditional hierarchical culture of the medical system [52, 53].

Confirming the results of previous studies [22, 24, 26, 30, 31, 54], participants regularly reported difficulties in addressing PPC services to families due to the strong (perceived) stigma surrounding this word. To overcome this obstacle many participants adopted a euphemistic term, such as comfort care, supportive care or *accompagnement*. This finding confronts us with the following paradox: given that the definition of PPC has evolved considerably over the last decades and its *ascribed* (formal) meaning in the guidelines is highly beneficent (“improvement of life quality”), why then is the *lay* meaning still overly negative [1, 2, 22]? Prior studies suggest that negative attitudes towards PPC among patients and families are often influenced by staff members’ own negative image of PPC [25, 32, 33, 36]. Some of our participants seemed to confirm these findings and this might confirm the idea that like many Western societies, Swiss society is still death-denying. Still we believe that given the centrality of death and dying in the Swiss public debate, it is difficult to maintain that this topic is still taboo. Hence, it is crucial to better understand care providers’ own perception of PPC to comprehend their aversion of the term palliative care. Our results show that various participants personally disliked the term “palliative care” not, or at least not primarily, because of its strong association with death or dying (many of them, in fact, were in favour of understanding PC as non-curative!) but because they considered it to be a concept that is incompatible with children’s everyday life. In this context, it is interesting to reflect further on the meaning of the terms, “support”, “comfort” and “*accompagnement*”. They are all words that are widely used in daily life to describe a willingness to be with others, to stand by someone, to be present [55]. This ordinariness stands in sharp contrast with the term “palliative care” which was perceived by the providers as being too policy-loaded. This finding is worth exploring more fully as it can provide novel insights into the conceptual barriers impeding implementation of PPC and thus be used to frame the design and analysis of future empirical studies on this topic. Although we cannot address this topic extensively here, we should keep in mind that both on an institutional level and in the public debate, PC is often discussed and promoted as a life- and choice-affirming alternative to euthanasia and physician assisted suicide as PC intends neither to hasten nor postpone death and patient preferences are heavily promoted in the WHO definition of PC [6, 56]. This means that PC is often debated within a context of autonomy and choice in which planning towards and acceptance of death are actively encouraged. This may explain why some participants considered the concept of PC to be out of touch with children’s perspective. By using synonyms such as comfort, support and “*accompagnement*” they might have wanted to reinforce PC’s original aim of being close to patients and families in care and affection.

Some participants were skeptical about re-branding PPC. They insisted on endorsing the term and debunking its negative meanings by introducing PPC earlier. In this way families will be less scared to revise the treatment goals the moment their child is not doing well. Other participants, however, critized this integrative approach. They argued that holistic care *is* provided at the time of diagnosis and wondered what the added value would be of introducing PPC at the start of the illness trajectory. In line with other studies, the question that we have to ask is: if PPC is no longer a special type of care, but becomes part of the medical “mainstream”, independent of any advanced prognosis, then does it not become too de-coupled from death [2]? Some authors are concerned that in order to improve acceptance, PPC guidelines have become death-sensitive and risk marginalizing those for whom PC was developed in the first place: dying children and their bereaved families [57–60]. A few participants seemed to share this worry when they reflected on the limited bereavement care possibilities in pediatric oncology.

A small number of participants addressed the possibility of rebranding PPC by relying on positive word-of-mouth. Studies have shown that word-of-mouth communication might have a great influence on the healthcare behaviour of the general public, especially in our current era of internet-based communication and the increasing use of social media [61].

A last concern expressed by the participants was the interaction with patients and families from a different, non-Swiss cultural and religious background. At present, there is little or no research on how ethnic and religious minorities perceive and understand PC definitions [62].

### Limitations

First, because of the mixed focus group approach and the possible power differences that go along with it, it is possible that not all participants freely expressed their views on the subject matter, so some issues might have been left unsaid. Furthermore, the familiarity among the participants (most of them were part of the same team) might have resulted in taken-for granted “party-line” attitudes regarding PPC [63]. However, we intentionally used such an approach to mirror the actual “natural” group dynamics in the Swiss SPOGs where PPC is usually provided by the primary oncology team, which includes nurses, psychologists, psycho-oncologists etc. in addition to physicians. This inter-professionalism might have allowed the production of insights that could have been less accessible in single provider type groups. Moreover the moderators encouraged all participants to engage actively during the debate. Finally, the advantage of using an acquaintance group was that participants could provide more details regarding certain events or experiences and challenge the statements of other participants if they did not correspond to the actual hospital practice [63]. Second, given the specific Swiss pediatric oncology context, findings are not generalizable to other contexts abroad with specialist PPC teams and a different healthcare system. Third, since the focus group data collection was part of a bigger project that has been running since 2012, a few of the study participants knew the research project and the team already. Thus, we cannot exclude that the responses of some participants might have been influenced by their perception of the overall goal of the project. Finally, because most of our participants were women, our findings might be gender biased. However, since the workforce in both the pediatric and palliative care context is predominantly female, our sample does reflect the setting that we wanted to examine.

## Conclusion

Despite important changes in the formal definition of PPC, it has still a overly negative connotation in the minds of many parents and healthcare providers. To counter this trend, calls have been made to initiate PPC at diagnosis and if necessary, rebrand the term PPC as supportive or comfort care. Many participants in this study seemed critical about the “from diagnosis onward” directive and clearly associated PPC with non-curative treatment. To most of them, the adequate timing of PPC remained a major challenge. Although the philosophical definition of PPC leaves room for patient individuality, it complicates clinical practice as it does not provide clear protocols. More referral tools are needed to help oncologists to identify children and families with palliative care needs. Further, although PPC has increasingly profiled itself as being concerned with the patient’s quality of life (rather than with death) this shift has not overcome all stigmas. Therefore, perhaps the conceptual obstacle to PPC is not so much death itself, but the way in which PC is discussed on both a policy level and in public debates, that is, in terms of choice, autonomy and personal development. This interpretation could find support in the fact that our participants considered the term “palliative care” to be out of touch with the child’s perspective and preferred to use synonyms that are closer to PC’s original aims: to offer support to patients and families in pain, anger, sadness and laughter without any normative expectations. Other well-designed empirical studies are needed to further explore these findings. Still, although the use of these alternative terms might be a useful and pragmatic approach to overcoming the *initial* stigma [30], in the end, it might be ineffective as these words might gradually acquire the same negative connotations as PC as long as there is no change in the public discourse on dying. The best way to counter this trend may be to promote (on- and offline) positive word-of-mouth [64] among satisfied families and health care providers. By sharing their stories, families and healthcare providers could become the true ambassadors of PPC. More research is needed on how healthcare professionals can use online word-of-mouth on social media for PPC advocacy. Also, further efforts should be pursued to develop PPC educational and training programs for healthcare staff (including conscious self-reflection). Finally, critical reflection is needed on the possible practical and conceptual shortcomings in PPC guidelines themselves, in order to better support PPC healthcare providers.

## Data Availability

Anonymized quotes taken from the interview transcripts are directly available within this publication. Additional raw data (full transcripts) related to this publication cannot be openly released due to ethical constraints.

## Abbreviations

PC: Palliative care
PPC: Pediatric palliative care
SPOG: Swiss pediatric oncology group centers
WHO: World Health Organization

## Footnotes

1 Switzerland is a federal state which is divided into 26 cantons. Each canton has its own cantonal constitution, approved by the federal parliament. For the current study, we obtained the approval of 4 research ethics committees.

2 It is difficult to adequately translate the French word “accompagnement” in English since it is deeply indebted into the Francophone culture. The notion emphasizes patients’ embeddedness in society, rather than putting the focus on their individuality. The best translation would maybe be “accompanying”, “to be a companion”.
